# Prevalence of Allergen-Specific IgE Positivity and Serum Immunoglobulin E Concentrations of Allergens in Dogs with Suspected Allergic Dermatitis Using the Multiple Allergen Simultaneous Test in South Korea

**DOI:** 10.3390/vetsci12060563

**Published:** 2025-06-08

**Authors:** Yoon-Seok Jang, Jae-Il Han, Eun-Soo Lee, Doo-Sung Cheon, Aryung Nam, Jae-Eun Hyun

**Affiliations:** 1 Department of Veterinary Internal Medicine, College of Veterinary Medicine, Konkuk University, Seoul 05029, Republic of Korea; nwind12@konkuk.ac.kr (Y.-S.J.); zaet123@konkuk.ac.kr (J.-I.H.); kyma310@konkuk.ac.kr (E.-S.L.); aryung@konkuk.ac.kr (A.N.); 2 Postbio, Gyeonggido 12106, Republic of Korea

**Keywords:** canine atopic dermatitis, dog, food allergy, immunoglobulin E, multiple allergen simultaneous test

## Abstract

Type I allergies are triggered by immunoglobulin E (IgE)-mediated hypersensitivity reactions upon allergen exposure. Dogs are diagnosed with allergic dermatitis based on history, clinical signs, and allergen-specific IgE detection. Using the multiple allergen simultaneous test (MAST)–immunoblot assay, this study measured IgE concentrations and analyzed the proportion of dogs showing allergen-specific IgE positivity, and IgE concentrations of environmental and food allergens in South Korea. We examined data from canine serum using the MAST assay in 2023; the allergen panel included 130 allergens. Data were analyzed, with results greater than zero regarded as positive for the prevalence measurements and concentrations compared among subgroups. Overall, 2663 samples were evaluated to assess the proportion of dogs showing allergen-specific IgE positivity and mean concentrations of environmental and food allergens. Among the environmental allergens, *Alternaria* spp. had the highest IgE prevalence, whereas Japanese cedar had the highest mean IgE concentration. Allergen-specific differences were observed among subgroups categorized by age, sex, and breed. To our knowledge, this research is the first large-scale study to analyze canine serum using a MAST assay to assess the IgE prevalence of allergen-specific IgE positivity and concentration and to examine data by age, sex, and breed. These findings provide information for diagnosis and management of canine allergies.

## Simple Summary

In dogs, specific substances, such as environmental factors, foods, and drugs, can trigger allergies, leading to excessive immune responses and antibody generation caused by exposure to allergens. This study investigated allergen-specific immunoglobulin E (IgE) levels in the serum of dogs suspected of having allergic dermatitis in South Korea, aiming to explore regional, age, sex, and breed-related differences in sensitization to environmental and food allergens. Overall, *Alternaria* spp. was found to be the most prevalent environmental allergen, while lamb meat was the most prevalent food allergen. The highest IgE concentrations were observed in Japanese cedar for environmental allergens and flaxseed for food allergens. Additionally, this study analyzed the allergens predominantly found in South Korea and the IgE concentration of sensitization related to these allergens by age, sex, and breed. These findings provide valuable data for minimizing allergen exposure and customizing immunotherapy to improve the management of allergy symptoms in dogs.

## 1. Introduction

Type I allergy is an IgE-mediated hypersensitivity reaction triggered by specific substances known as allergens, which can be present in the environment, foods, and drugs [1]. Serum immunoglobulin E (IgE) plays a key role in the adaptive immune system and mediates hypersensitivity reactions upon allergen exposure [2].

Type I allergy includes both CAD (Canine Atopic Dermatitis) and FAs (Food Allergies), depending on the sensitizing allergen [1]. In dogs, CAD is a genetically predisposed inflammatory skin condition associated with IgE antibodies primarily directed against environmental allergens [3,4], while FAs involve an immune response to specific food proteins [5]. Both are common dermatological conditions. An allergy is initially characterized by pruritus, which manifests as behaviors such as scratching, licking, rubbing, and head shaking, with lesions commonly occurring on the face, concave aspect of the pinnae, ventrum, axillae, inguinal region, perineal area, and distal extremities [3]. Identifying allergen-specific IgE in conjunction with clinical symptoms and a medical history is valuable for the diagnosis of allergic diseases [6]. When skin lesions are diagnosed as being caused by an allergy, elevated serum IgE levels are associated with the severity of allergic disease symptoms [7]. In contrast to CAD, FAs exhibit limited correlation between serum IgE levels and clinical manifestations, making elimination and provocation trials the preferred methods for accurate diagnosis [8,9]. Although strict dietary inclusion or exclusion criteria were not applied, food allergens were included in the present study to investigate sensitization patterns and to provide supplementary epidemiological insights within the study population.

In dogs exhibiting clinical signs suggestive of allergic skin disease, the diagnosis of allergic skin conditions can be supported by detecting allergen-specific IgE in serum [10]. The measurement of allergen-specific IgE has been developed based on various principles, including radioimmunoassay, enzyme immunoassay, and immunoblot techniques [6]. Accordingly, several assay methods are available, such as the radioallergosorbent test (RAST), the enzyme-linked immunosorbent assay (ELISA), and the multiple allergen simultaneous test (MAST). The MAST-immunoblot assay is widely used because, compared to the RAST and the ELISA, it enables the simultaneous detection of multiple allergens using a small volume of serum through a relatively rapid procedure, thereby reducing the financial burden on pet owners [1,6]. As a result, the increasing use of the MAST-immunoblot assay by clinicians may contribute to a more comprehensive understanding of allergen-specific IgE sensitization profiles. Nevertheless, no large-scale studies have evaluated allergen-specific IgE detection using the MAST in dogs.

Hence, this study aimed to evaluate the associations between elevated serologic IgE levels—induced by environmental and food allergens—and dog subgroups categorized by age, sex, and breed in South Korea, according to the MAST assay results.

## 2. Materials and Methods

### 2.1. Data Collection

Blood samples were collected from dogs presenting with suspected allergic dermatitis at regional veterinary hospitals across South Korea and were forwarded to the laboratory (PobaniLab, South Korea) of one of the co-authors for diagnostic analysis between January and December 2023. The serum samples used in this study were originally submitted for the purpose of diagnosing allergic skin disease. Patient information, including age, sex, and breed, was obtained from the submitted request forms.

This study adhered to the Animal Protection Act of the Republic of Korea and the relevant animal welfare guidelines. Serum samples from dogs were collected as part of routine diagnostic procedures by licensed veterinarians to ensure appropriate animal handling and to minimize stress. Informed consent for blood collection and for the use of diagnostic results was obtained from the owners prior to clinical examination through a signed owners’ consent form.

### 2.2. Sample Collection and Transportation

At the veterinary hospital, 1–2 mL of serum was collected from each blood sample and aliquoted into sterile tubes according to the laboratory’s instructions. These serum samples were immediately shipped to the laboratory in a cooler bag (4–10 °C) and tested within 2 h of arrival.

### 2.3. MAST–Immunoblot Assay

The EuroBlotOne system (Euroimmun, Lübeck, Germany) and the RoboScreen™ system (Mediwiss Analytic GmbH, Moers, Germany) were used for the MAST–immunoblot assay according to the manufacturers’ instructions, and all other experimental conditions were consistently maintained throughout the analyses. Briefly, 300 μL of each patient’s serum was pipetted into a reaction chamber containing allergens immobilized on a nitrocellulose membrane and then incubated at room temperature for 45 min. After washing, the serum was added to an anti-canine IgE antibody conjugated with biotin, followed by incubation at room temperature for 30 min. This biotin-conjugated antibody acts as a secondary antibody, catalyzing the transformation of a specific substrate with distinguishable properties. After washing to remove the unbound antibodies, the serum was added to 300 μL of streptavidin conjugated to alkaline phosphatase and then incubated at room temperature for 20 min. The unbound conjugate was removed by washing. Next, we added the color-development solution, incubated the sample at room temperature for 20 min, and then dried the strips. The results were converted into IU/mL using a formula, specific to each antigen, which was built into the program based on the band’s color intensity [1]. All subjects underwent a MAST for 130 allergens classified into eight groups (molds, mites, insects, trees, grasses, weeds, others, and foods) as determined by the laboratory. Table S1 in the Supplementary Materials lists the specific allergens in each group.

### 2.4. Data and Statistical Analysis

Serum samples from dogs with suspected allergic dermatitis were analyzed using a standardized testing protocol that included both environmental and food allergen panels to identify overall sensitization patterns.

The data reflecting elevated allergen-specific IgE concentrations were transferred to a Microsoft Excel database (Microsoft Corporation, Redmond, WA, USA) [11]. We calculated the prevalence of allergen-specific IgE positivity as the proportion of nonzero values and defined the mean IgE concentration as the average of all values greater than zero. The mean IgE concentration is expressed as the 95% confidence interval (CI) and standard deviation (SD). For comparison, the data were categorized by age, sex, and breed. We used Fisher’s exact test and the one-way analysis of variance for analyzing the prevalence and the mean IgE concentration, respectively [12,13]. Each breed was evaluated relative to the entire group using the chi-square test [12] for prevalence and the *t*-test [14] for mean IgE concentration. Statistical data were analyzed using the IBM SPSS (version 28.0; IBM Corp., Armonk, NY, USA) and the SAS (version 9.4; SAS Institute Inc., Cary, NC, USA). A *p*-value < 0.05 was considered statistically significant. We used GraphPad Prism (version 10.4.1; GraphPad Software, San Diego, CA, USA) to visualize the overall prevalence of allergen-specific IgE positivity and mean IgE concentrations of environmental and food allergens [15] and to compare sex-based differences.

## 3. Results

### 3.1. Analysis of the Prevalence of Allergen-Specific IgE Positivity and Mean IgE Concentrations of Major Environmental Allergens Using Serologic Allergen-Specific IgE Test Results

**Figure 1:**
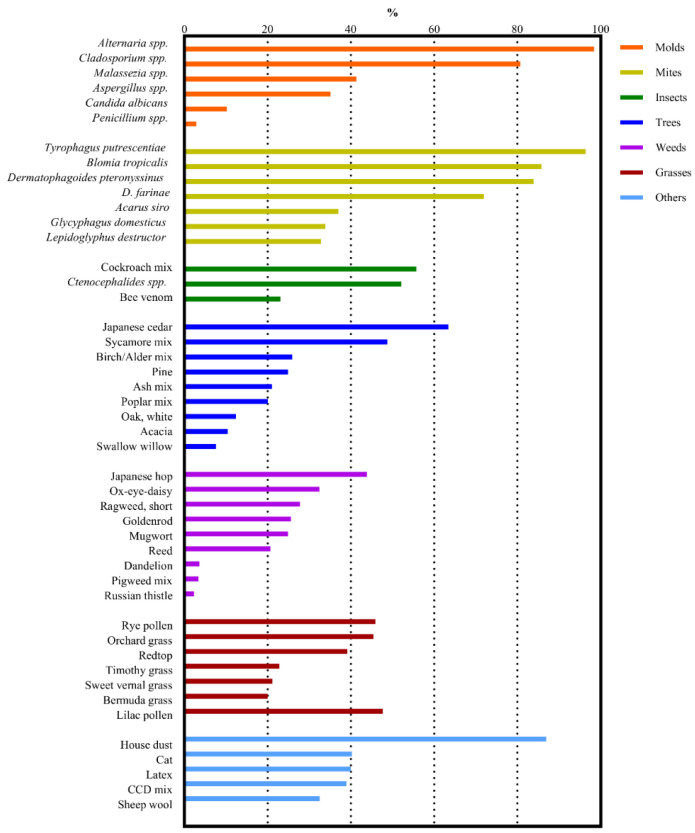
Prevalence of allergen-specific IgE positivity of allergens within environmental allergen groups (molds, mites, insects, trees, weeds, grasses, and others) analyzed through serologic IgE testing, presented in descending order.

**Figure 2:**
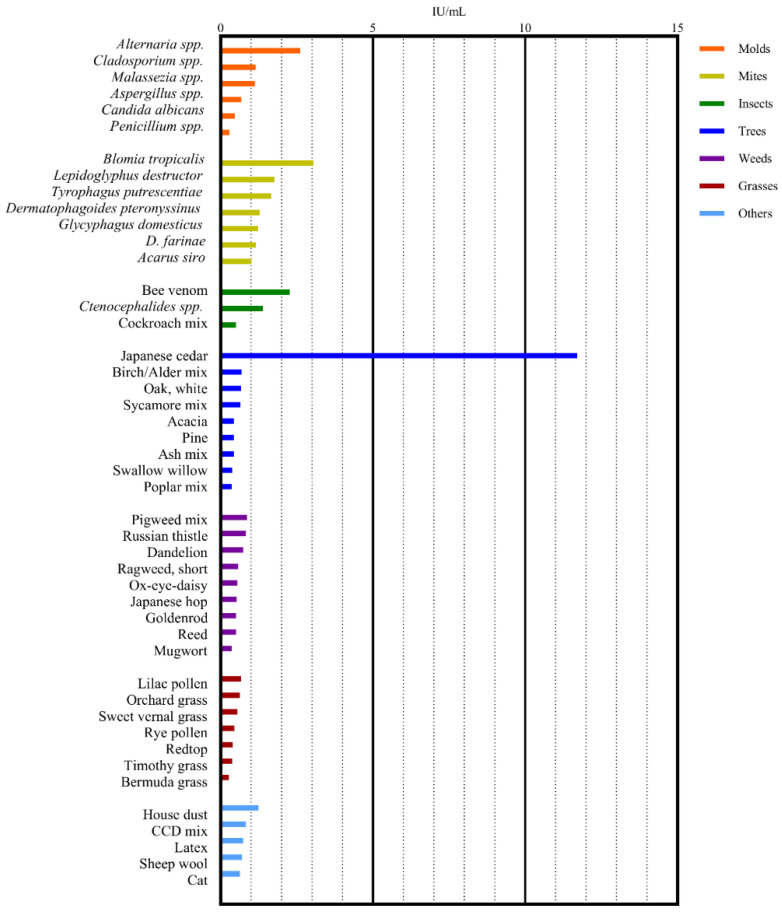
Mean IgE concentrations of allergens within environmental allergen groups (molds, mites, insects, trees, weeds, grasses, and others) examined through serologic IgE testing, sorted in descending order.

This study analyzed serologic allergen-specific IgE test results from 2663 dogs that tested positive for at least one allergen. Table 1 lists the signalments of the enrolled dogs. The allergen-specific IgE was most frequently detected in canine serum when exposed to the environmental allergen *Alternaria* spp., followed by *Tyrophagus putrescentiae*, house dust, *Blomia tropicalis*, *Dermatophagoides pteronyssinus*, *Cladosporium* spp., *D. farinae*, Japanese cedar, cockroach mix, and *Ctenocephalides* spp. Mites accounted for four of these allergens, molds and insects for two each, and trees and others for one each. The canine serum showed the highest concentrations of allergen-specific IgE upon exposure to the allergen Japanese cedar, followed by *B. tropicalis*, *Alternaria* spp., bee venom, *Lepidoglyphus destructor*, *T. putrescentiae*, *Ctenocephalides* spp., *D. pteronyssinus*, house dust, and *Glycyphagus domesticus*. Table 2 presents the prevalence of allergen-specific IgE positivity and the mean serum IgE concentrations of the 10 most common environmental allergens. Figure 1 and Figure 2 illustrate such prevalence of allergen-specific IgE positivity and mean IgE concentration by allergen group.

### 3.2. Comparison of the Prevalence of Allergen-Specific IgE Positivity and Mean Serum IgE Concentrations of Environmental Allergens According to Age

The age at testing was between 2 months and 17.1 years (median: 4.3 years). The dogs were categorized according to age [16]: puppies (<1 year), adolescents (1 to <3 years), young adults (3 to <7 years), older adults (7 to <11 years), and seniors (11+ years). Among the 10 most prevalent environmental allergens, *Alternaria* spp., *B. tropicalis*, *D. pteronyssinus*, *Cladosporium* spp., and *Ctenocephalides* spp. showed an increasing prevalence with age. However, *T. putrescentiae* and *D. farinae* were less prevalent in seniors than in older adults. Japanese cedar was less prevalent in older adults than in young adults. Furthermore, house dust and cockroach mixtures were less prevalent among adolescents and older adults than among puppies and young adults.

The mean IgE concentration of Japanese cedar decreased with age (*p* = 0.003), whereas that of *Alternaria* spp. increased (*p* < 0.001). *T. putrescentiae* and *Ctenocephalides* spp. produced the highest mean IgE concentrations in young adults, with statistical significance. Table 3 presents the statistical significance of the prevalence of allergen-specific IgE positivity and mean IgE concentration comparisons among the five age groups.

### 3.3. Prevalence of Allergen-Specific IgE Positivity and Mean Serum IgE Levels of Environmental Allergens According to Sex

The dogs were categorized into five categories as follows: intact males, neutered males, intact females, neutered females, and unknown. However, the comparisons excluded the unknown group. Among the 10 most prevalent allergens, house dust, and *Ctenocephalides* spp. were higher in intact males, whereas *B. tropicalis* was higher in neutered dogs than in intact dogs. However, the prevalence of these allergens did not significantly differ among subgroups within the sex categories (Supplementary Materials, Figure S1).

For the mean IgE concentrations of the top-ranking allergens, *T. putrescentiae* (*p* = 0.007) was significantly higher in intact and neutered females than in intact and neutered males.

### 3.4. Comparison of the Prevalence of Allergen-Specific IgE Positivity and Mean IgE Concentrations of Environmental Allergens Between the Overall Group and the Five Specific Breeds

In this study, serum samples obtained for testing were categorized by breed to compare allergen-specific IgE sensitization patterns, aiming to identify potential breed-related predispositions and to examine the associated clinical and epidemiological tendencies. The serologic IgE test analysis included 36 dog breeds. In the breed classification, the “Mix” category refers to dogs resulting from the crossbreeding of two officially recognized breeds (e.g., Maltese and Poodle). Since these dogs cannot be classified as a single breed, they were excluded from the breed-specific subgroup. The five most frequently tested breeds were Bichon Frisé (17.2%), Maltese (16.5%), Toy Poodle (15.6%), Pomeranian (7.3%), and Shih Tzu (3.2%), accounting for 60.4% of the total. In these breeds, *Alternaria* spp. and *T. putrescentiae* consistently ranked first and second in prevalence, respectively.

In the five most frequently tested breeds, Japanese cedar exhibited the highest mean IgE concentration. Among the other groups, mite allergens were consistently among the most prevalent. Certain allergens showed notably elevated levels in specific breeds, although they were not similarly elevated in other breeds; for example, cat allergen in Malteses, Russian thistle in Pomeranians, and pigweed mix (*p* = 0.013) in Shih Tzus. Table 4 presents the 10 most prevalent environment allergens and their mean IgE concentration according to the breed.

### 3.5. Food Allergen Evaluation Based on the Results of Allergen-Specific Serologic IgE Testing

The allergen-specific IgE was most frequently detected in canine serum when exposed food allergens such as lamb meat, followed by flaxseed, pineapple, brewer’s yeast, corn, watermelon, quail, spinach, aloe vera, and beta-lactoglobulin (Supplementary Materials, Table S2). The prevalence of allergen-specific IgE positivity had a SD of 0.24, indicating relatively small differences among them. Moreover, the concentration of allergen-specific IgE was high in dogs, especially when exposed to the allergen flaxseed, followed by aloe vera, lamb meat, corn, plum, pineapple, casein, watermelon, brewer’s yeast, and gluten (Supplementary Materials, Table S3).

The serologic IgE prevalence of allergen-specific IgE positivity for food allergens increased with age, except among the 10 frequently detected food allergens, where older adults showed a lower prevalence than young adults. This difference was statistically significant for lamb meat, flaxseed, pineapple, brewer’s yeast, corn, watermelon, quail, and beta-lactoglobulin. The mean IgE concentrations based on age differed by allergen; for instance, aloe vera and plum resulted in a decrease with age, whereas brewer’s yeast caused an increased.

Moreover, the prevalence of allergen-specific IgE positivity showed no significant difference within the sex-based subgroups, but neutered males and females tended to have a higher prevalence than intact males and females. However, in the neutered females, lamb meat (*p* = 0.003) and casein (*p* < 0.001) gave the highest mean IgE concentrations.

The most prevalent food allergens were similar among the five most frequently tested breeds, although peach and cacao allergies were more common in Shih Tzus. The mean IgE concentrations of the major food allergens were also consistent across breeds, with flaxseed producing the highest concentrations in all breeds.

## 4. Discussion

This study analyzed the prevalence of allergen-specific IgE positivity and mean IgE concentrations of allergens in 2663 canine serum samples, which were refrigerated and collected in South Korea between January 1 and December 31, 2023. Previous studies have reported that nonfrozen serum may yield higher sensitivity in IgE detection [17]; however, all samples in the present study were stored under refrigerated conditions and were not frozen. The present study employed the MAST assay, which is known for its accuracy comparable to the ELISA and its ability to quickly and simultaneously analyze multiple allergens [1]. Although the sensitivity and specificity may be lower compared to the SPT, a MAST has the advantages of being applicable regardless of the patient’s skin condition and reduced stress imposed on the patient [18]. Among environmental allergens, *Alternaria* spp. had the highest prevalence, consistent with the findings of a previous small-scale study conducted at a regional hospital in South Korea [19]. *Alternaria* spp. is pathogenic to both humans and animals, and its year-round presence in both indoor and outdoor environments may significantly impact allergic responses [20].

House dust mites are the primary allergens responsible for CAD worldwide [21]. However, in our study, *T. putrescentiae*, a storage mite, was the most predominant among mite allergens, followed by house dust mites such as *B. tropicalis*, *D. pteronyssinus*, and *D. farinae*. A previous study on environmental allergens in humans in South Korea identified *D. pteronyssinus* and *D. farinae* as the most prevalent mite allergens, contrary to our findings [22]. In this study, the prevalence of the flea allergen *Ctenocephalides* spp. was lower than that of house dust mites and storage mites. This finding is consistent with previous studies conducted in Italy and other European regions, where the prevalence of flea allergens has also been reported to be moderate to low [4]. Allergic reactions to fleas differ from those to other allergens in terms of hypersensitivity mechanisms, and the possibility of cross-reactivity with other allergens exists. These factors may have influenced the results [23,24]. Therefore, ongoing research on regional allergen sensitization in dogs may provide key insights to enhance diagnostic accuracy and improve the effectiveness of allergen-specific immunotherapy in managing canine allergic skin diseases.

Environmental allergens can be further classified into indoor and outdoor allergens [4]. An Italian study that employed the ELISA identified grass allergens, including Bermuda grass and timothy grass, as the most common outdoor allergens in dogs; sheep sorrel was also frequently detected. Similarly, a study in South Korea using the MAST-immunoblot assay reported a high prevalence of grass allergens in humans [22]. However, in our study, the most common outdoor allergens in dogs were tree allergens such as Japanese cedar, sycamore mix, and pine rather than grass allergens. Considering methodological differences, our interpretation aimed to identify overarching patterns, with the observed variation potentially stemming from differences in exposure resulting from geographical and climatic factors affecting outdoor allergen distribution [4]. Another possible reason is the differences in sensitization rates between dogs and humans [25]. Further large-scale longitudinal studies using consistent analytical methods are needed to investigate allergen prevalence and temporal trends in response to regional and environmental variations in veterinary medicine.

The rankings of the prevalence of allergen-specific IgE positivity and mean serum IgE concentrations of allergens differed in this study. Japanese cedar had the highest mean IgE concentration among the environmental allergens, followed by *B. tropicalis* and *Alternaria* spp. In humans, IgE antibody concentrations specific to *Alternaria* spp. peak during adolescence and early adulthood but sharply decline after the age of 25–27 years [26]. Conversely, in dogs, IgE levels specific to *Alternaria* spp. increased continuously with age. Previous human studies reported an association between serologic IgE concentrations and the severity of allergic symptoms, as well as their role in increasing the risk of allergy in asymptomatic individuals [27,28], suggesting that comparable immunologic mechanisms may also be involved in dogs. Serum IgE concentrations are useful not only for selecting allergens for allergen-specific immunotherapy but also for monitoring treatment responses [7,29]. Accordingly, the findings of this study may provide valuable insights into regional and age-related sensitization patterns and contribute to the formulation of effective immunotherapy strategies.

This study also compared the prevalence of allergen-specific IgE positivity and IgE concentrations of allergens across age, sex, and breed groups. Ten environmental allergens, that are widely distributed in South Korea, showed no significant differences according to sex or neuter status, consistent with previous findings [30,31,32]. However, the age-based analysis showed that the 10 allergens with relatively high occurrence were less prevalent in puppies and adolescents than in young adults, older adults, and seniors. This finding aligns with a previous South Korean study that analyzed the prevalence of allergen-specific IgE using an ELISA kit [33]. However, in our study, *Alternaria* spp. prevalence across age groups was statistically insignificant. One reason for this result is the unbalanced distribution of the data, particularly the very low number of “negative” cases in the senior group, with only one case. To improve the balance of data distribution, we need to conduct further research with a larger sample size to increase the number of negative cases.

The five breeds most frequently tested in this study (Bichon Frisé, Maltese, Toy Poodle, Pomeranian, and Shih Tzu) are not among the commonly reported high-risk breeds for CAD [12]. However, the breed-specific analysis of IgE concentrations revealed notable differences. Cat allergens were elevated in the Maltese, and Russian thistle and pigweed were present in the Pomeranian and Shih Tzu breeds, respectively, compared with other breeds. The clinical significance of these elevated IgE concentrations in certain breeds remains unclear. Hence, the prevalence, concentration, and potential associations between allergen-specific IgE and clinical symptoms in various breeds requires further investigation.

In dogs living in Australia, Europe, and North America, food elimination and provocation trials have demonstrated that beef, chicken, and wheat flour are the most frequently identified food allergens [34]. However, our study did not observe clear differences in food allergen prevalence or identify dominant food allergens. The prevalence of beef, chicken, and wheat flour was relatively low, which is inconsistent with the findings of a previous study conducted in South Korea during a similar period [19]. Instead, the most prevalent food allergens in this study were flax seed, lamb meat, corn, and pineapple, all of which showed high serum IgE concentrations. This discrepancy may be attributed to the short half-life of IgE and its susceptibility to recent exposure to food allergens [9]. As serologic IgE testing alone cannot confirm food allergies and may reflect recent exposure rather than true sensitization, its results should be interpreted with caution. Therefore, to confirm food allergies in dogs, additional diagnostic methods such as elimination diets and provocation trials are necessary. Serologic testing may serve as a supportive tool in guiding the development of an appropriate treatment plan. Moreover, the results of the present study showed that Shih Tzus had a higher IgE positivity rate to peach and to cacao compared to other breeds, and elevated IgE concentrations to flaxseed were observed across all breeds. These findings suggest the possibility of breed-specific sensitization to certain allergens as well as regional sensitization patterns, highlighting the need for further research to clarify these associations.

This study has some limitations that warrant consideration. First, clinical information such as skin lesions, respiratory symptoms, gastrointestinal symptoms, and diet-related history was not included. Although allergen sensitization is a significant risk factor for the development of allergic diseases, it does not necessarily indicate clinical allergy, as some sensitized individuals may remain asymptomatic [28]. Allergen-specific IgE testing using the MAST assay is known to have high sensitivity but low specificity; nevertheless, it remains a useful screening tool for identifying clinically suspected cases [10]. Future studies should aim to more precisely evaluate the diagnostic utility and clinical relevance of allergen-specific IgE testing by correlating the results with clinical signs and intradermal test outcomes and dietary history.

Second, this study analyzed data from a single year, which limited our ability to assess annual, seasonal, or monthly variations in serum IgE levels. In addition, the absence of both negative and positive control groups presents a limitation in evaluating the diagnostic validity of the findings. Future longitudinal multi-year studies incorporating appropriate control groups could help identify trends in pollen allergies, temporal changes in allergen prevalence, and fluctuations in mean IgE concentrations. Such studies would also aid in developing strategies to minimize allergen exposure in dogs with elevated IgE levels and enhance the diagnostic robustness of allergen-specific IgE testing [35,36].

## 5. Conclusions

This study is the first large-scale analysis of canine serum that uses the MAST assay to investigate the prevalence of allergen-specific IgE positivity and serum IgE concentrations of allergens. Using this assay, we measured allergen-specific IgE levels across environmental and food allergens. The data were further examined according to age, sex, and breed, providing insights into variations within canine populations. The findings of this study may assist clinicians in early decision-making for dietary management and allergen-specific immunotherapy by providing population-level IgE sensitization patterns. The emphasis on sensitivity enhances its utility as a screening tool in clinical settings.

## Figures and Tables

**Table 1:** Demographic distribution of dogs undergoing multiple allergen simultaneous tests in 2023.

Category			Number of Dogs (%)
Sex	Male	Intact male	112 (4.2)
		Neutered male	1301 (48.9)
	Female	Intact female	198 (7.4)
		Neutered female	1005 (37.7)
	Unknown		47 (1.8)
Age	Puppies (<1 year)		268 (10.1)
	Adolescents (1 to <3 years)		893 (33.5)
	Young adults (3 to <7 years)		913 (34.3)
	Older adults (7 to <11 years)		472 (17.7)
	Seniors (11+ years)		117 (4.4)
Breed	Bichon Frisé		472 (17.7)
	Maltese		440 (16.5)
	Toy Poodle		416 (15.6)
	Pomeranian		195 (7.3)
	Shih Tzu		85 (3.2)
	Chihuahua		63 (2.4)
	French bulldog		59 (2.2)
	Coton de Tulear		48 (1.8)
	Yorkshire Terrier		43 (1.6)
	Mix		452 (17.0)

**Table 2:** Prominent allergens were identified according to their prevalence of allergen-specific IgE positivity and mean allergen-specific IgE concentrations in canine serum.

Rank	Environmental Allergens	Percentage (%)	95% CI (%)	Environmental Allergens	Mean IgE Concentration (IU/mL)	95% CI (IU/mL)
1	*Alternaria* spp.	98.4	97.8–98.8	Japanese cedar	11.71	11.21–12.20
2	*Tyrophagus putrescentiae*	96.4	95.6–97.0	*B. tropicalis*	3.04	2.93–3.16
3	House dust	86.9	85.6–88.2	*Alternaria* spp.	2.62	2.52–2.70
4	*Blomia tropicalis*	85.8	84.5–87.1	Bee venom	2.27	1.69–2.84
5	*Dermatophagoides pteronyssinus*	83.9	82.5–85.3	*Lepidoglyphus destructor*	1.77	1.52–2.03
6	*Cladosporium* spp.	80.7	79.2–82.2	*T. putrescentiae*	1.66	1.58–1.75
7	*D. farinae*	71.9	70.1–73.5	*Ctenocephalides* spp.	1.38	1.05–1.20
8	Japanese cedar	63.4	61.6–65.2	*D. pteronyssinus*	1.28	1.17–1.40
9	Cockroach mix	55.7	53.8–57.6	House dust	1.24	1.14–1.35
10	*Ctenocephalides* spp.	52.1	50.2–54.0	*Glycyphagus domesticus*	1.22	1.17–1.28

CI: confidence interval.

**Table 3:** Age-related comparison of environmental allergen-specific IgE prevalence of allergen-specific IgE positivity and mean concentrations in dogs according to the MAST results.

	Rank	Allergens	Puppies	Adolescents	Young Adults	Older Adults	Seniors	*p*-Value
Prevalence (%)	1	*Alternaria* spp.	97.4	97.9	98.7	99.2	99.2	0.217
	2	*Tyrophagus putrescentiae*	94.4	95.6	96.9	98.1	95.7	0.041
	3	House dust	86.6	83.3	89.2	89.0	89.7	0.003
	4	*Blomia tropicalis*	81.0	84.3	87.1	87.7	91.5	0.016
	5	*Dermatophagoides pteronyssinus*	79.5	81.0	85.5	87.7	88.9	0.001
	6	*Cladosporium* spp.	74.3	76.7	81.9	86.4	94.0	<0.001
	7	*D. farinae*	66.0	68.2	74.4	76.9	73.5	0.001
	8	Japanese cedar	56.7	61.9	65.6	64.0	70.9	0.029
	9	Cockroach mix	56.3	51.3	58.5	56.6	62.4	0.016
	10	*Ctenocephalides* spp.	44.0	52.4	53.2	53.4	54.7	0.088
Mean IgE concentration (IU/mL)	1	Japanese cedar	13.14	12.46	11.58	10.66	8.77	0.003
	2	*B. tropicalis*	2.93	2.98	3.18	3.00	2.93	0.577
	3	*Alternaria* spp.	2.25	2.42	2.73	2.85	2.92	<0.001
	4	Bee venom	1.52	1.92	2.79	1.59	4.35	0.256
	5	*Lepidoglyphus destructor*	1.47	1.67	1.97	1.75	1.44	0.786
	6	*T. putrescentiae*	1.25	1.44	1.87	1.87	1.73	<0.001
	7	*Ctenocephalides* spp.	1.14	1.34	1.59	1.23	1.20	0.030
	8	*D. pteronyssinus*	0.99	1.23	1.37	1.35	1.32	0.421
	9	House dust	1.18	1.23	1.24	1.20	1.72	0.414
	10	*Glycyphagus domesticus*	1.18	1.23	1.26	1.19	1.19	0.896

**Table 4:** Breed-specific comparison of major environmental allergens with the highest prevalence of allergen-specific IgE positivity and mean IgE concentrations according to serologic IgE analysis in dogs.

	Rank	Allergens	Bichon Frisé	Allergens	Maltese	Allergens	Toy Poodle	Allergens	Pomeranian	Allergens	Shih Tzu
Prevalence (%)	1	*Alternaria* spp.	98.3	*Alternaria* spp.	99.1	*Alternaria* spp.	97.8	*Alternaria* spp.	97.4	*Alternaria* spp.	97.7
	2	*Tyrophagus putrescentiae*	95.3	*T. putrescentiae*	96.1	*T. putrescentiae*	96.9	*T. putrescentiae*	95.9	*T. putrescentiae*	95.3
	3	*Blomia tropicalis*	85.4	House dust	87.7	House dust	87.3	House dust	90.3	*Cladosporium* spp.	85.9
	4	House dust	84.5	*D. pteronyssinus*	86.1	*B. tropicalis*	86.1	*B. tropicalis*	83.1	House dust	85.9
	5	*Dermatophagoides* *pteronyssinus*	80.9	*B. tropicalis*	85.7	*D. pteronyssinus*	82.5	*D. pteronyssinus*	82.1	*D. pteronyssinus*	83.5
	6	*Cladosporium* spp.	77.5	*Cladosporium* spp.	82.5	*Cladosporium* spp.	81.3	*Cladosporium* spp.	79.0	*B. tropicalis*	81.2
	7	*D. farinae*	67.2	*D. farinae*	71.4	*D. farinae*	75.0	*D. farinae*	63.6	*D. farinae*	78.8
	8	Japanese cedar	62.3	Japanese cedar	65.7	Japanese cedar	58.9	Japanese cedar	63.1	Japanese cedar	64.7
	9	Cockroach mix	54.2	Cockroach mix	57.7	Cockroach mix	51.9	Cockroach mix	59.0	Cockroach mix	60.0
	10	*Ctenocephalides* spp.	53.0	Sycamore mix	50.9	*Ctenocephalides* spp.	48.3	*Ctenocephalides* spp.	49.2	*Ctenocephalides* spp.	58.8
Mean IgE concentration (IU/mL)	1	Japanese cedar	11.57	Japanese cedar	10.79	Japanese cedar	12.65	Japanese cedar	12.79	Japanese cedar	11.34
	2	*B. tropicalis*	2.90	*B. tropicalis*	2.92	*B. tropicalis*	3.03	Russian thistle	5.06	Pigweed mix	3.90
	3	*Alternaria spp.*	2.51	*Alternaria* spp.	2.65	*Alternaria* spp.	2.39	*B. tropicalis*	3.11	Bee venom	3.37
	4	Bee venom	2.09	*T. putrescentiae*	1.78	*L. destructor*	1.77	*Alternaria* spp.	2.45	*B. tropicalis*	2.74
	5	*Lepidoglyphus destructor*	1.60	Bee venom	1.70	*T. putrescentiae*	1.56	*T. putrescentiae*	1.41	*Alternaria* spp.	2.57
	6	*T. putrescentiae*	1.44	*L. destructor*	1.55	*D. pteronyssinus*	1.26	*Ctenocephalides*	1.36	*T. putrescentiae*	2.24
	7	*Ctenocephalides* spp.	1.43	*D. pteronyssinus*	1.31	*Ctenocephalides* spp.	1.20	*L. destructor*	1.30	*L. destructor*	1.96
	8	House dust	1.37	*Ctenocephalides* spp.	1.26	Bee venom	1.13	*G. domesticus*	1.20	*Acarus siro*	1.95
	9	*D. pteronyssinus*	1.36	Cat	1.18	*Malassezia* spp.	1.13	House dust	1.18	*D. pteronyssinus*	1.66
	10	*Glycyphagus domesticus*	1.25	*G. domesticus*	1.14	*G. domesticus*	1.11	*Malassezia* spp.	1.12	*D. farinae*	1.61

## Data Availability

The data presented in this study are available upon request from the corresponding author.

## Supplementary Materials

The following supporting information can be downloaded at: https://www.mdpi.com/article/10.3390/vetsci12060563/s1, Table S1: Classification of allergen panels used in the MAST assay into environmental and food allergens; Table S2: The allergen-specific IgE prevalence for food allergens in canine serum, analyzed using the MAST assay and classified by age, sex, and breed; Table S3: The mean concentrations of allergen-specific IgE for food allergens in canine serum, analyzed using the MAST assay and classified by age, sex, and breed; Figure S1: (A) Sex-related prevalence of environmental allergen-specific IgE in dogs based on the MAST results. (B) Sex-related mean concentrations of environmental allergen-specific IgE in dogs based on the MAST results.

## Abbreviations

IgE	immunoglobulin E
CAD	canine atopic dermatitis
FAs	food allergies
RAST	radioallergosorbent test
ELISA	enzyme-linked immunosorbent assay
MAST	multiple allergen simultaneous test
CI	confidence interval
SD	standard deviation
