# Supplementary material for: Prevalence of Allergen-Specific IgE Positivity and Serum Immunoglobulin E Concentrations of Allergens in Dogs with Suspected Allergic Dermatitis Using the Multiple Allergen Simultaneous Test in South Korea
Source: Vet Sci. 2025 Jun 8;12(6):563. doi: 10.3390/vetsci12060563 (PMC12197348; doi:10.3390/vetsci12060563)
Supplement: Supplementary file 1 [file vetsci-12-00563-s001.zip › Figure S1.pdf]

Figure S1: (A) Sex-related prevalence of environmental allergen-specific IgE in dogs based on the MAST results. (B) Sex-related mean concentrations of environmental allergen-specific IgE in dogs based on the MAST results.

(A) Sex-related prevalence of environmental allergen-specific IgE in dogs based on the MAST results.

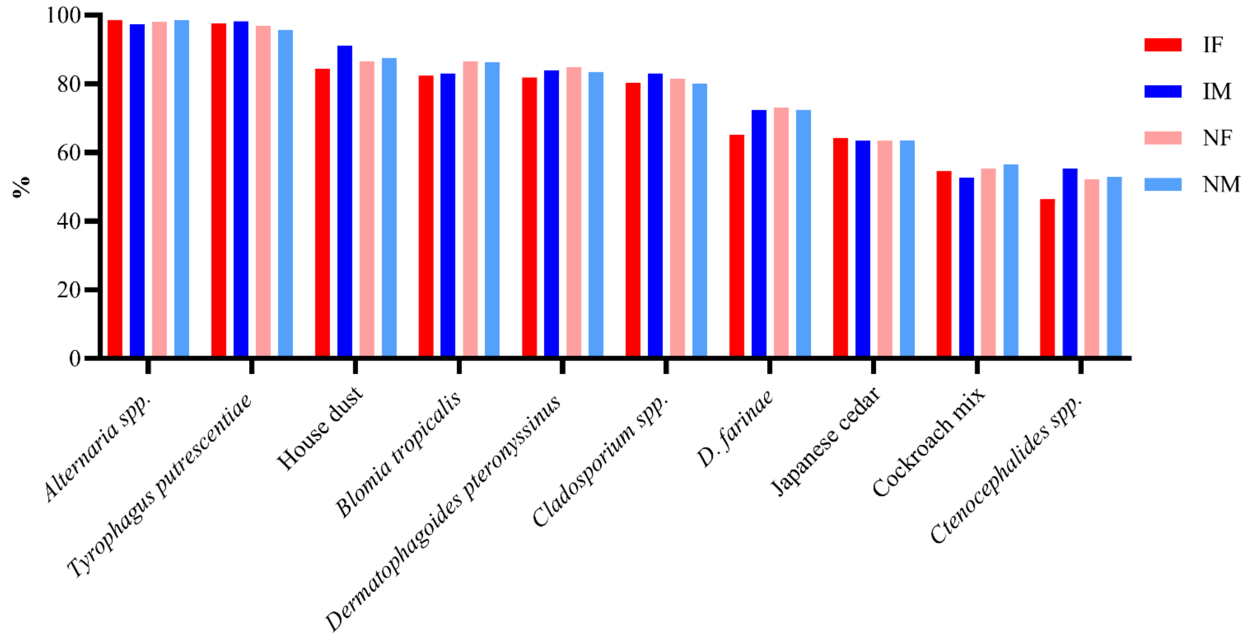

(B) Sex-related mean concentrations of environmental allergen-specific IgE in dogs based on the MAST results.

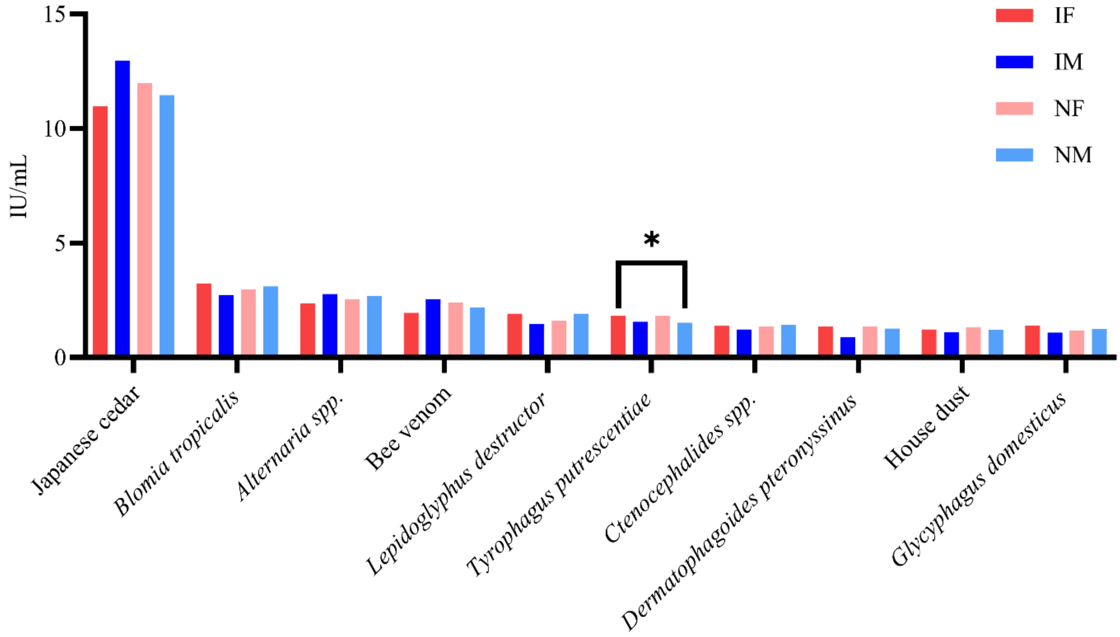

IF, intact female; IM, intact male; NF, neutered female; NM, neutered male
The asterisk indicates a p-value < 0.05
